# UNC5B Promotes Vascular Endothelial Cell Senescence via the ROS-Mediated P53 Pathway

**DOI:** 10.1155/2021/5546711

**Published:** 2021-06-20

**Authors:** Zhen Yang, Han Li, Pengcheng Luo, Dan Yan, Ni Yang, Yucong Zhang, Yi Huang, Yu Liu, Le Zhang, Jinhua Yan, Cuntai Zhang

**Affiliations:** Department of Geriatrics, Tongji Hospital, Tongji Medical College, Huazhong University of Science and Technology, Wuhan, Hubei 430030, China

## Abstract

Vascular endothelial cell senescence is involved in human aging and age-related vascular disorders. Guidance receptor UNC5B is implicated in oxidative stress and angiogenesis. Nonetheless, little is known about the role of UNC5B in endothelial cell senescence. Here, we cultured primary human umbilical vein endothelial cells to young and senescent phases. Subsequently, the expression of UNC5B was identified in replicative senescent cells, and then, its effect on endothelial cell senescence was confirmed by UNC5B-overexpressing lentiviral vectors and RNA interference. Overexpression of UNC5B in young endothelial cells significantly increased senescence-associated *β*-galactosidase-positive cells, upregulated the mRNAs expression of the senescence-associated secretory phenotype genes, reduced total cell number, and inhibited the potential for cell proliferation. Furthermore, overexpression of UNC5B promoted the generation of intracellular reactive oxygen species (ROS) and activated the P53 pathway. Besides, overexpression of UNC5B disturbed endothelial function by inhibiting cell migration and tube formation. Nevertheless, silencing UNC5B generated conflicting outcomes. Blocking ROS production or inhibiting the function of P53 rescued endothelial cell senescence induced by UNC5B. These findings suggest that UNC5B promotes endothelial cell senescence, potentially by activating the ROS-P53 pathway. Therefore, inhibiting UNC5B might reduce endothelial cell senescence and hinder age-related vascular disorders.

## 1. Introduction

Cardiovascular and cerebrovascular disorders immensely contribute to the global health and economic burden [1]. Epidemiological studies reveal that advanced age is a major risk factor for cardiovascular and cerebrovascular diseases [2, 3]. Moreover, emerging evidence indicates that shared molecular mechanisms of vascular aging underlie the pathogenesis of age-related macrovascular and microvascular diseases [3]. Vascular endothelial cells residing in the innermost layer of vascular tissue occupy a crucial position in vascular health. Therefore, establishing the molecular mechanisms underlying endothelial cell senescence is necessary.

Similar to most cellular senescence, endothelial cell senescence program is triggered by extracellular and intracellular stresses like telomere shortening, oxidative stress, DNA damage, and epigenetic changes [4]. These stressors could be interrelated and engage various downstream effector pathways but ultimately activate P53, P16, or both [5, 6]. Among them, oxidative stress, triggered by the accumulation of the reactive oxygen species (ROS), is a primary mechanism underlying the cellular senescence process [7] and promotes age-associated endothelial dysfunction [8]. Despite moderate ROS levels being essential for normal functions in cells, overproduction of ROS causes deleterious effects, including DNA damage. In response to DNA damage, the P53-dependent pathway is activated causing a P21-dependent cell cycle arrest [9]. Specifically, significant evidence demonstrates that senescent endothelial cells are dysfunctional exhibiting age-related impairment of angiogenesis [10–12]. Age-related impairment of angiogenesis is considered to be one of the primary factors causing increased morbidity and mortality of vascular disease [13]. Accordingly, understanding the mechanisms of oxidative stress in age-related endothelial cell senescence can help to address the increased risk of age-related vascular disorders.

Guidance receptors have been demonstrated to not only participate in axonal sprouting in the nervous system but also take part in the formation and remolding of the vascular systems [14]. Moreover, guidance receptors regulate the progression of neurological and cardiovascular disorders [14]. Transmembrane receptor UNC5B is one of the vascular-specific axon guidance receptors whose expression is detected in various tissues, specifically in endothelial cell clusters [15–18]. Dysregulation of UNC5B expression in macrophages is implicated in atherosclerosis development and plaque stability [19]. Nonspecific repression of UNC5B significantly attenuated myocardial ischemia-reperfusion injury [18]. As such, UNC5B might be involved in the development of age-related disease. Although UNC5B has been demonstrated to be related to angiogenesis in endothelial cells [20], their relationship with aging remains unreported. A recent study has shown that UNC5B might be implicated in the generation of intracellular ROS induced by ultraviolet-B irradiation exposure [21]. However, the regulatory role of UNC5B alone in oxidative stress and other biological processes is unclear. Besides, UNC5B was also reported to act as a tumor suppressor and mediate P53-dependent apoptosis [22–24]. Since senescence is conventionally a potent tumor suppressor mechanism, UNC5B could contribute to cellular senescence.

The parabiotic model supports the idea that transferable factors in blood rescue age-related degenerative phenotypes [25–28]. Our preliminary research indicated that UNC5B participated in restoring endothelial cell senescence and age-related endothelial dysfunction with young human plasm. To evaluate our hypothesis, i.e., UNC5B regulates endothelial cell senescence, a prototypical model of replicative cellular senescence was used in primary human umbilical vein endothelial cells (HUVECs). We demonstrated that UNC5B was induced in replicative senescent endothelial cells and overexpression of UNC5B accelerated senescence. Thereafter, since oxidative stress is a primary factor of cell senescence, and P53 modulation potentially influencing both senescence and apoptosis, this work investigated if senescence triggered by UNC5B could be attributed to the ROS-P53 pathway. Eventually, we examined the impacts of UNC5B on age-related vascular function.

## 2. Materials and Methods

### 2.1. Isolation and Culture of Endothelial Cells

Primary HUVECs were obtained from five different human umbilical cords, as previously described [29]. The collection of human umbilical cords was approved by the Ethics Committee for Human Experimentation of Tongji Hospital. Isolated HUVECs were cultured in complete Medium 199 supplemented with 10% FBS (Biological Industries, Israel) and Low Serum Growth Supplement (Gibco, USA). HUVECs were passed with 0.05% trypsin-EDTA (Gibco, USA) at intervals of every 2-3 days. The population doubling level (PDL) was obtained based on previously described formulas [30]. Replicative senescent cells were generated by consecutively passaging the young HUVECs until the cell proliferation was nearly arrested, which was approximately at PDL 29 but varied from one individual to another. Young and senescent cells used in the experiments were cells from PDL 5 to 13 and PDL 25 to 33, respectively.

### 2.2. Senescence-Associated-*β*-Galactosidase (SA-*β*-gal) Assay

The SA-*β*-gal activity was measured using Senescence Cells Histochemical Staining Kit (Sigma, USA) based on the manufacturer's instructions. Briefly, cells were incubated in the 1X Fixation Buffer at room temperature for 6-7 min then stained with the Staining Mixture at 37°C without CO_2_ overnight. Subsequently, cells were observed and visualized under a light microscope. The blue-stained cells and the total number of cells were counted using ImageJ (NIH, USA).

### 2.3. RNA Extraction and Quantitative RT-PCR (qPCR) Analysis

Total RNA was purified by RNA purification kit (Magen, China) and reverse-transcribed using the ReverTra Ace® qPCR RT kit (TOYOBO, Japan) following the manufacturer's instructions. SYBR® Green Realtime PCR Master Mix (TOYOBO, Japan) was applied in determining the mRNA levels of different genes using the primers listed in Table 1 on an ABI Step One Plus (Applied Biosystems, USA). GAPDH was used as an endogenous control.

### 2.4. Western Blot

Total protein was lysed in RIPA buffer (Boster, China) containing a protease inhibitor cocktail (Boster, China). Protein lysates were separated by 8-12% sodium dodecyl sulfate-polyacrylamide gel electrophoresis then transferred onto polypropylene difluoride membranes (Millipore, USA). The membranes were blocked in 5% nonfat milk at room temperature for 1 hour and incubated with the following antibodies: UNC5B (1 : 1000; Cell Signaling Technology, USA), P53 (1 : 1000; R&D, USA), P21 (1 : 500; Santa Cruz, USA), and GAPDH (1 : 5000; Proteintech, China) at 4°C overnight. After washing 3 times in 1X TBST (Tris-buffered saline containing 0.1% Tween 20), the membranes were incubated with respective secondary antibodies conjugated with horseradish peroxidase (1 : 5000; Promotor, China) at room temperature for 1 hour. Eventually, the membranes were rewashed three times with TBST and visualized using an enhanced chemiluminescence kit (Beyotime, China).

### 2.5. RNA Interfering

Small interfering RNA- (siRNA-) targeting UNC5B and negative control (NC) were designed and synthesized by RiboBio (RiboBio, China). Replicative senescent HUVECs were transfected with the siRNAs using Lipofectamine™ 3000 (Thermo Fisher, USA). Cells were seeded in a complete medium approximately 16 hours before transfection. Subsequently, siRNA mixed with Lipofectamine™ 3000 was added to the cells with a fresh 2% FBS medium. siRNAs were transfected at a concentration of 50 nM. After 6 hours, the medium containing siRNAs and Lipofectamine™ 3000 was replaced with a complete medium.

### 2.6. Lentiviral Overexpression of UNC5B

Lentiviral production was purchased from the GENECHEM (Shanghai, China) and infected HUVECs at PDL5 following the manufacturer's instructions. HUVECs were infected at a multiplicity of infection of 100 with control lentiviral vector or UNC5B-overexpressing lentiviral vector in the presence of HitansG P for 8 hours and designed as “Ctrl” or “UNC5B OE”, respectively. Puromycin selection was initiated (500 ng/ml) after 3 days. qPCR and western blotting were used to detect the expression level of UNC5B in transfected cells.

### 2.7. Cell Proliferation Assay

Edu incorporation assay was used to detect cell growth by directly measuring DNA synthesis using iClick™ EdU Andy Fluor 555 Imaging Kit (GeneCopoeia, USA) following the manufacturer's instructions. Following cell staining, five random fields were counted and photographed under a microscope. The ImageJ software was used in the quantification of Edu-positive and DAPI-positive cells. CCK-8 test was used to monitor growth as an indirect measure for proliferation. Transfected cells were seeded in 96-well plates at a similar density and incubated for 0, 24, 48, or 72 hours. Using a SUNRISE-microplate reader, the number of viable cells after treatment with CCK-8 was evaluated by measuring the absorbance at 450 nm.

### 2.8. Measurement of Reactive Oxygen Species (ROS)

Intracellular ROS generation was evaluated via a fluorescence microscope using dihydroethidium (DHE) fluorescent dye (MCE, China) based on the manufacturer's instructions. Briefly, HUVECs were washed with M199 and stained with DHE (10 *μ*mol/L) at 37°C with 5% CO_2_ for 30 min. After washing 3 times with M199, ethidium fluorescence was observed and photographed under an inversion fluorescence microscope.

### 2.9. Immunofluorescence

*γ*-H2AX protein expression in transfected HUVECs was detected using immunofluorescence. The cells were grown on coverslips and adequately fixed with 4% paraformaldehyde. After washing with PBS, cells were blocked in PBS with 5% goat serum and 0.5% Triton-100X at room temperature for one hour. Then, cells were incubated with primary antibody *γ*-H2AX (1 : 100 dilution, Cell Signaling Technology, USA) at 4°C overnight. After washing with PBS, the cells were incubated with fluorescently tagged secondary antibodies (1 : 500 dilution, Cell Signaling Technology, USA) at room temperature for 1 hour, followed by an anti-fluorescence quencher containing DAPI to seal the sections and identify nuclei. The cells were visualized and imaged using a Nikon C2 confocal microscope.

### 2.10. Endothelial Tube Formation and Migration Assays

The endothelial tube formation assay was performed using HUVECs. Growth factor-reduced Matrigel (Corning, USA) was spread into 96-well culture plates (50 *μ*l/well) then allowed to rest at 37°C for 1 hour. Subsequently, transfected cells were suspended and counted using a Cellometer-Mini Automatic Cell Counter (Nexcelom Bioscience, Lawrence, MA). Cells were seeded at the density of 5000 cells/well on the Matrigel-coated plates and incubated at 37°C with 5% CO_2_ for 6 hours. The network formation was photographed using an inverted light microscope. The degree of tube formation was quantified using the plugin “Angiogenesis Analyzer” from the ImageJ software.

The migration ability of HUVECs was evaluated with a transwell assay. Briefly, cells were serum-starved overnight and then seeded in the upper compartment of a 24-well transwell plate at a density of 3 × 10^4^ cells (Corning, USA). A complete medium supplemented with 10% FBS was added to the bottom wells. HUVECs were incubated for 24 hours to allow them to migrate through the 8.0 *μ*m polycarbonate membrane. Thereafter, cells on the lower surface of the membrane were fixed and stained with crystal violet staining solution (0.1% crystal violet, 20% methanol in PBS). The migrated cells were observed under a microscope. To quantify the signals, the bound dye was solubilized with 95% ethanol solution and the absorbance was measured at 570 nm.

### 2.11. Statistical Analysis

Statistical analyses were performed using the IBM SPSS software (version 22.0). Data were described as mean ± standard error of the mean (SEM) and analyzed by Student's *t*-test, two-way analysis of variance (ANOVA), or one-way ANOVA. A two-sided *P* < 0.05 was considered statistically significant.

## 3. Results

### 3.1. Upregulation of UNC5B in Senescent Endothelial Cells

**Figure 1:**
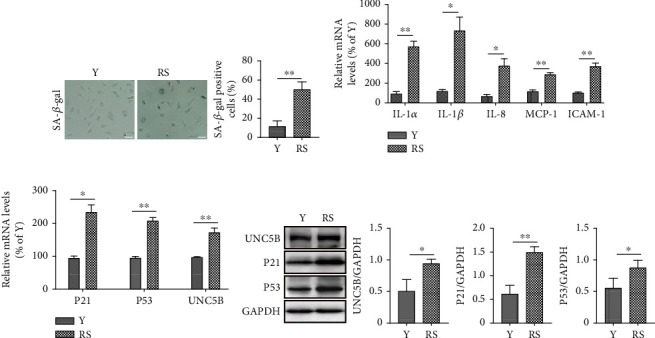
The expression of UNC5B is induced in senescent endothelial cells. Senescence-associated markers were used to characterize senescent endothelial cells. (a) Representative images of SA-*β*-gal staining assay in young (Y) and replicative senescent (RS) HUVECs. The positive cell quantification of the two groups is shown. (b) Fold changes in expression levels of the SASP mRNAs (*IL-1α*, *IL-1β*, *IL-8*, *MCP-1*, and *ICAM-1*) in senescent cells compared to young cells. (c) *P21*, *P53*, and *UNC5B* mRNA expression in young and senescent cells. (d) The protein expression of P21, P53, and UNC5B in young and senescent cells determined by western blot analysis. Scale bar, 50 *μ*m. Quantitative data are presented as mean ± SEM of three or more independent experiments. ^∗^ means *P* < 0.05; ^∗∗^ means *P* < 0.01.

To estimate the role of UNC5B during senescence *in vitro*, a replicative senescent cell model was established in primary HUVECs. The SA-*β*-gal assay and the senescence-associated secretory phenotype genes (SASP) mRNAs were used as senescence-associated markers for identifying senescence. Consequently, unlike young cells (PDL 5-13), replicative senescent cells (PDL 25-33) demonstrated a higher proportion of blue staining (Figure 1(a)) and a significant increase in all tested SASP mRNAs including *IL-1α*, *IL-1β*, *IL-8*, *MCP-1*, and *ICAM-1* (Figure 1(b)). Meanwhile, both the mRNA and protein levels of UNC5B were significantly upregulated in senescent endothelial cells, similar to P21 and P53 (Figures 1(c) and 1(d)). Collectively, these findings indicate that UNC5B was upregulated in senescent endothelial cells.

### 3.2. Downregulation of UNC5B Ameliorates Endothelial Cell Senescence

**Figure 2:**
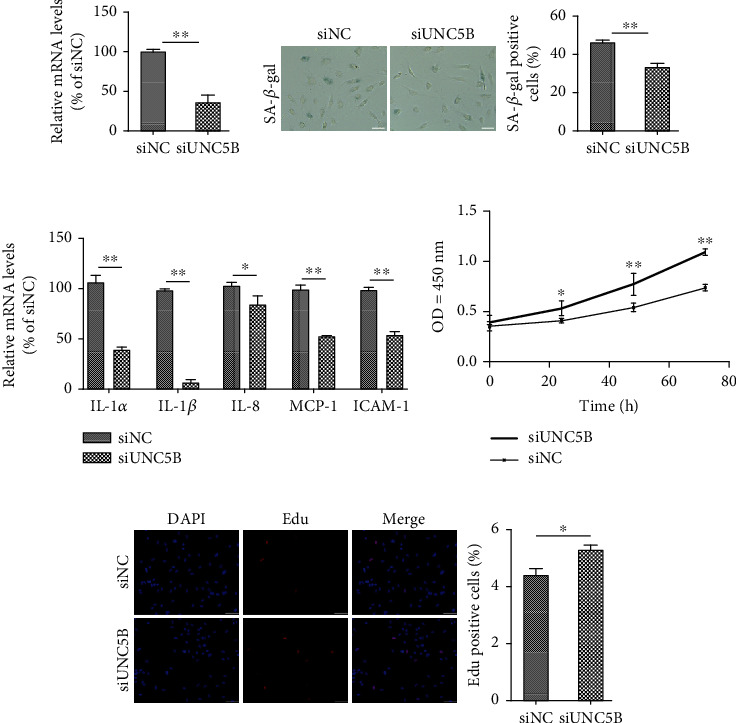
Downregulation of UNC5B ameliorates the senescence-associated phenotype. Replicative senescent HUVECs were transfected with negative control (NC) or siRNA UNC5B. (a) Silence efficacy confirmed by detecting the mRNA level of *UNC5B*. (b) Representative images of SA-*β*-gal staining assay and the percentage rate of SA-*β*-gal positive cells. (c) SASP evaluated by analyzing the mRNA expression levels of *IL-1α*, *IL-1β*, *IL-8*, *MCP-1*, and *ICAM-1*. (d) Growth curve analysis in transfected cells at the indicated time points. (e) Number of proliferative cells as detected by the Edu incorporation assay. Scale bar, 50 *μ*m. Quantitative data are presented as mean ± SEM of three or more independent experiments. ^∗^ means *P* < 0.05; ^∗∗^ means *P* < 0.01.

To clarify the function of UNC5B in endothelial cell senescence, expression of UNC5B was downregulated in replicative senescent endothelial cells (PDL 25-33) using RNA interference. The transcriptional level of *UNC5B* was knocked down approximately 70% after siRNA transfection (Figure 2(a)). The proportion of senescent cells was lower among UNC5B-silenced cells than among the control (Figure 2(b)). The mRNA expression of the SASP genes, i.e., *IL-1α*, *IL-1β*, *IL-8*, *MCP-1*, and *ICAM-1*, was significantly attenuated by inhibition of UNC5B (Figure 2(c)). Further, the proliferation of control and UNC5B-silenced cells was assessed. As a result, UNC5B-silenced cells proliferated at a faster rate than the control cells based on the growth curve analysis (Figure 2(d)) and Edu incorporation assay (Figure 2(e)). These results reveal that the downregulation of UNC5B ameliorated the senescence-associated phenotype.

### 3.3. Overexpression of UNC5B Induces Premature Senescence in Young Endothelial Cells

**Figure 3:**
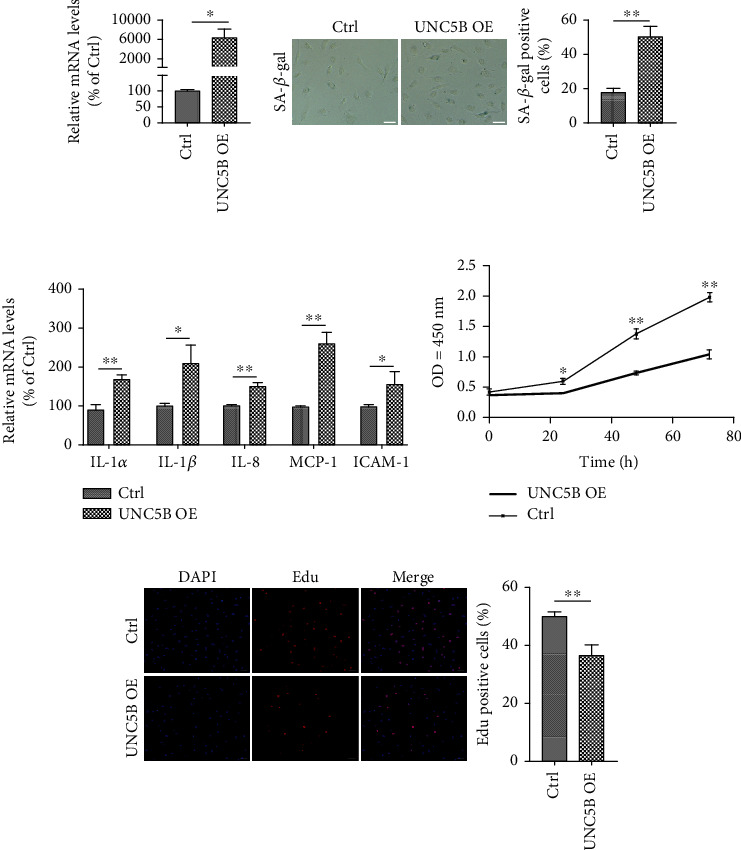
Overexpression of UNC5B promotes young endothelial cells undergoing premature senescence. Young HUVECs were infected with control lentiviral vectors (Ctrl) or UNC5B-overexpressing lentiviral vectors (UNC5B OE). (a) *UNC5B* mRNA levels confirmed in control cells and UNC5B-overexpressing cells. (b) The transfected cells seeded in 24-well plates for SA-*β*-gal staining and calculation of the percentage of positive cells. (c) Evaluation of SASP by analyzing the mRNA expression levels of *IL-1α*, *IL-1β*, *IL-8*, *MCP-1*, and *ICAM-1*. (d) Growth curve analysis in transfected cells at the indicated time points. (e) Number of proliferative cells as detected by the Edu incorporation assay. Scale bar, 50 *μ*m. Quantitative data are presented as mean ± SEM of three or more independent experiments. ^∗^ means *P* < 0.05; ^∗∗^ means *P* < 0.01.

To further explore the function of UNC5B in endothelial cell senescence, the effect of UNC5B overexpression on premature senescence was evaluated in young HUVECs (PDL 5-13). Overexpression of UNC5B was effectively achieved by lentivirus transfection (Figure 3(a)). UNC5B-overexpressing cells displayed an increase in the proportion of SA-*β*-gal-positive cells than control cells (Figure 3(b)). The mRNA expression of the SASP genes, i.e., *IL-1α*, *IL-1β*, *IL-8*, *MCP-1*, and *ICAM-1*, was also significantly upregulated in UNC5B-overexpressing cells compared to that of the controls (Figure 3(c)). Moreover, overexpression of UNC5B reduced total cell numbers according to growth curve analysis (Figure 3(d)) and inhibited cell proliferation potential as indicated by fewer Edu-positive cells (Figure 3(e)). These outcomes coincide with the above knockdown experiments and confirm that UNC5B is implicated in the progression of cellular senescence.

### 3.4. UNC5B Regulates Endothelial Cell Senescence in a ROS-Dependent Manner

**Figure 4:**
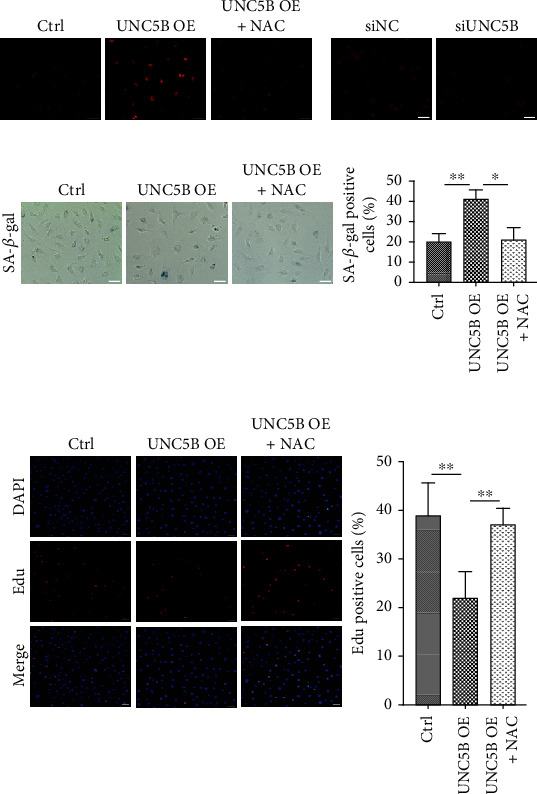
UNC5B induces endothelial cell senescence through ROS production. (a) Young HUVECs were prepared as Figure 3. The production of intracellular ROS was determined with DHE by microscopy. Representative images of stained cultures are shown. (b) Transfection of replicative senescent HUVECs with siRNA for three days. Determination of ROS levels in the cells. Representative images of stained cultures are shown. (c) Once infected with lentiviral vectors, cells were immediately incubated with or without NAC (5 mmol/L). The cells were then stained and analyzed for their SA-*β*-gal activity. (d) The proliferative potential of these cells treated with or without NAC was detected by Edu incorporation assay. Scale bar, 50 *μ*m. Quantitative data are presented as mean ± SEM of three or more independent experiments. ^∗^ means *P* < 0.05; ^∗∗^ means *P* < 0.01.

Increased ROS is one of the crucial mediators of cellular senescence. Dihydroethidium staining was performed to assess whether UNC5B produced ROS. UNC5B-overexpressing cells demonstrated greater red fluorescence than control cells (Figure 4(a)), indicating a higher level of intracellular ROS, whereas UNC5B-silencing cells showed contrasting results (Figure 4(b)). N-acetylcysteine (NAC), a ROS scavenger, effectively rescued the ROS level induced by UNC5B overexpression (Figure 4(a)). Then, the role of UNC5B-induced intracellular ROS production was explored in the induction of senescence. Consequently, UNC5B-overexpressing cells incubated with NAC presented with fewer senescent cells than that in the absence of NAC (Figure 4(c)). Moreover, an Edu incorporation assay indicated that relatively slow proliferation was observed in control UNC5B-overexpressing cells but not in NAC presence (Figure 4(d)). Therefore, UNC5B seemingly induced senescence by increasing the intracellular ROS level.

### 3.5. Cell Premature Senescence Induced by UNC5B in a P53-Dependent Manner

**Figure 5:**
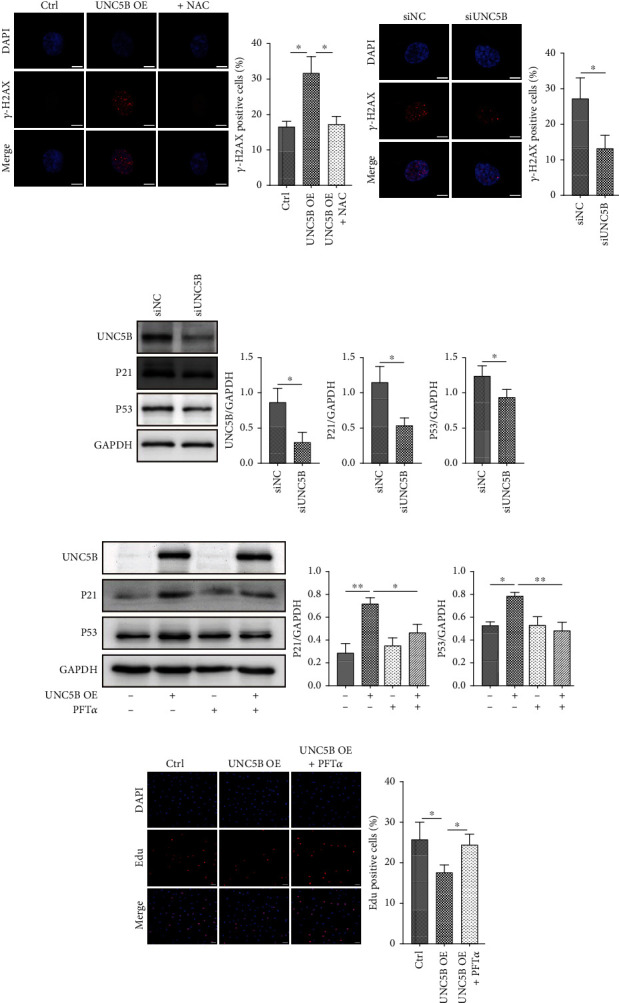
P53 is involved in UNC5B induced endothelial cell premature senescence. (a, b) Young control and UNC5B-overexpressing HUVECs or replicative senescent HUVECs transfection with siRNA for three days were subjected to immunofluorescence of *γ*-H2AX foci (red). Cells with no less than 20 foci were scored as *γ*-H2AX-positive cells. Approximately 150 cells were analyzed per experiment. Scale bar, 10 *μ*m. (c) Replicative senescent cells were transfected with siRNA for three days. The protein expression levels of UNC5B, P21, and P53 of the cells were analyzed by western blot. (d) After young cells infection with lentiviral vectors, PFT*α* or DMSO was added and maintained 5 *μ*mol/L in both control and UNC5B-overexpressing cells throughout the experiment. The cells were then lysed for western blot analysis. (e) The proliferative potential of these cells treated with or without PFT*α* was detected by Edu incorporation assay. Scale bar, 50 *μ*m. Quantitative data are presented as mean ± SEM of three or more independent experiments. ^∗^ means *P* < 0.05, ^∗∗^ means *P* < 0.01.

Increased levels of intracellular ROS trigger DNA damage. Therefore, the effect of ROS accumulation induced by UNC5B was monitored on *γ*-H2AX nuclear foci, an indicator of DNA damage. Immunofluorescence studies revealed that approximately 32% of UNC5B-overexpressing cells were highly positive (>20 foci per nuclei) for *γ*-H2AX nuclear foci against 17% in control cells (Figure 5(a)). Whereas ROS inhibition by NAC prevented the formation of *γ*-H2AX foci in UNC5B-overexpressing cells (Figure 5(a)). Levels of *γ*-H2AX foci-positive cells in UNC5B-silencing cells (13%) were lower than levels in control senescing cells (27%) (Figure 5(b)). Also, UNC5B-silencing cells showed downregulated protein expression levels of P53 and P21 (Figure 5(c)). For further clarification on the relationship between the P53 pathway and senescence induced by UNC5B, this work used PFT*α*, a P53-specific inhibitor. The presence of PFT*α* inhibited the upregulation of P53 induced by UNC5B overexpression and partially suppressed the expression of P21 (Figure 5(d)). Additionally, the presence of PFT*α* reversed the decrease in the percentage of Edu-positive cells induced by UNC5B overexpression (Figure 5(e)). Therefore, UNC5B could induce premature senescence through the ROS-P53 pathway in HUVECs.

### 3.6. UNC5B Induces Endothelial Dysfunction

**Figure 6:**
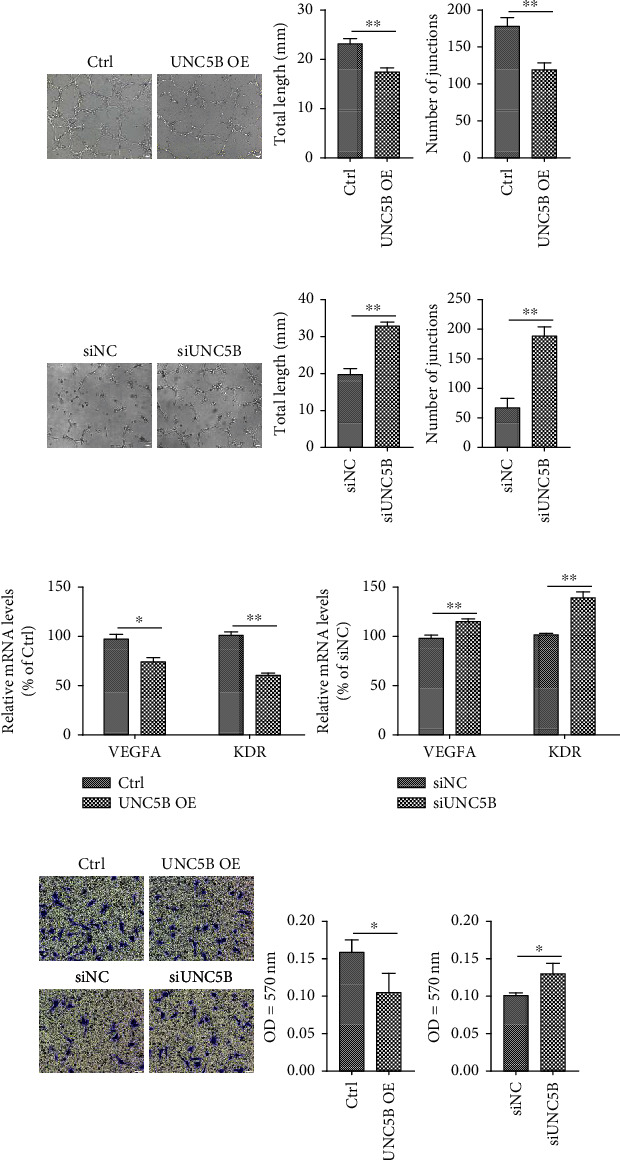
UNC5B impairs endothelial function. (a) In young HUVECs as prepared in Figure 3, the tube formation was observed in 96-well plates coated with Matrigel. (b) After replicative senescent cells transfection with siRNA for three days, the cells were cultured in Matrigel medium. Representative images of morphological changes of networks are shown. Lengths of the tubes and numbers of junctions were quantitated by the Image J software. (c) Angiogenic capacity evaluated by analyzing the mRNA expression levels of *VEGFA* and *KDR*. (d) Representative images of migration assay and its quantitative analysis. Scale bar, 50 *μ*m. Quantitative data are presented as mean ± SEM of three or more independent experiments. ^∗^ means *P* < 0.05, ^∗∗^ means *P* < 0.01.

Eventually, endothelial function was assessed through tube formation assay, angiogenic gene expression, and migration assay in the induction of senescence by UNC5B. For young endothelial cells, the control cells exhibited elongated and tubule-like structure, while UNC5B-overexpressing cells tube formation was hampered with an incomplete or sparse tubular network (Figure 6(a)). Inhibiting UNC5B in senescent cells increased the total length and number of junctions compared to control senescent cells (Figure 6(b)). Similarly, we found that the mRNA expression level of angiogenic genes, i.e., *VEGFA* and *KDR*, was significantly increased following UNC5B inhibition (Figure 6(c)). UNC5B-overexpressing cells exhibited decreased *VEGFA* and *KDR* mRNA levels (Figure 6(c)). The effect of UNC5B on HUVECs migration was evaluated using a transwell assay. Overexpression of UNC5B significantly impaired the migration of young cells (Figure 6(d)). Conversely, senescent cells exhibited better migratory capacity than control senescent cells following UNC5B knockdown (Figure 6(d)). These findings indicate that UNC5B disrupts endothelial function while inhibition of UNC5B enhances the vascular function.

## 4. Discussion

This study demonstrated that UNC5B is a novel regulator of senescence in vascular endothelial cells. By providing morphological and functional evidence, we showed that UNC5B induced endothelial cell senescence by inhibiting cell proliferation causing endothelial dysfunction by impairing tube formation and migration. Besides, endothelial cell premature senescence induced by UNC5B was primarily ROS-dependent and activated the P53 signaling pathway.

Notably, senescent endothelial cells are found in human and rodent arterial lesions involved in atherogenesis [31]. The present data confirmed that UNC5B was upregulated in senescent cells, confirming the findings of the Tampere Vascular Study, where UNC5B was upregulated in atherosclerotic plaques [19]. Nevertheless, Tampere Vascular Study failed to provide significant evidence for the positive correlation between UNC5B and endothelial cells, possibly due to the limited number of endothelial cells in atherosclerotic plaque.

Senescent cells characteristically exhibit high SA-*β*-gal activity and impaired proliferation. Previous studies revealed that UNC5B overexpression inhibits proliferation in human bladder cancer cells [32, 33]. Consistently, we noted that overexpression of UNC5B in young HUVECs increased SA-*β*-gal activity, decreased proliferation potential, and induced the expression of cyclin-dependent kinase inhibitory proteins P53 and P21. These findings indicate that UNC5B upregulation induces premature senescence, with a possible role in cell growth blockade. Interestingly, we found UNC5B inhibition significantly prevented endothelial cell senescence, demonstrated by decreased SA-*β*-gal activity, increased cell proliferation, and downregulated expression of P53 and P21. In line with these data, Yang et al. reported that inhibiting the activation of the UNC5B signaling pathway in vascular endothelial cells triggered a significant increase in cell proliferation [34]. Additionally, the above study demonstrated that knockdown of UNC5B without Netrin-1 intervention did not enhance cell proliferation. This was potentially attributed to the unachieved activation of UNC5B without Netrin-1 under the reported conditions. In line with this hypothesis, UNC5B should be activated when its expression is elevated to a certain level, as observed in overexpression and in senescent cells.

Age-related increase in ROS promotes endothelial cell senescence and endothelial dysfunction [8, 35]. This increase triggers a vicious accumulation cycle of DNA damage causing further activation of ROS production, thereby, more DNA damage [35, 36]. Recent reports indicate that inhibition of UNC5B blocked the inhibitory effect of exogenous netrin-1 on oxidative stress [37], suggesting a negative effect of netrin-1/UNC5B signaling on oxidative stress. Notably, Netrin-1 was not expressed in HUVECs [38]. For the first time, we report that UNC5B regulates oxidative stress independently of Netrin-1 during senescence. Knockdown of UNC5B in senescent cells inhibited the generation of ROS, overexpression of UNC5B induced the production of ROS, while NAC treatment attenuated SA-*β*-gal activity and enhanced proliferation in UNC5B-overexpressing cells. As such, the mechanism of UNC5B-induced senescence was at least partly via the production of ROS. Considering the absence of exogenous netrin-1, the regulation role of UNC5B alone on oxidative stress might be incompatible with the combined effects of Netrin-1 and UNC5B. Moreover, we noted that UNC5B activated a DNA damage response (DDR) as shown in *γ*-H2AX foci assay and such activation was attenuated by NAC. These findings suggest a possible involvement of the DNA damage pathway in UNC5B-induced senescence.

Importantly, our results demonstrated that overexpression of UNC5B in young endothelial cells significantly activated the P53/P21 signaling pathway, while inhibition of UNC5B in senescent cells suppressed the expression of P53 and P21. UNC5B gene is a direct transcriptional target for P53 and can be activated by multiple stresses in a p53-dependent manner [23]. Previous studies showed that UNC5B mediated P53-dependent apoptosis in the absence of netrin-1 [22]. Although the mechanisms between aging and apoptosis remain unclarified, studies have speculated that apoptosis contributes to aging [39]. Besides, accumulating evidence shows that P53 plays a critical role in senescence and apoptosis. Moreover, the persistence of DDR ultimately activates P53 which triggers either senescence or apoptosis pathways via stimulation or repression of various downstream targets transcription [40–42]. Therefore, we examined whether the P53 pathway was implicated in the regulation of senescence by UNC5B. The present data revealed that the upregulated expression of P53 and P21 in UNC5B-overexpressing cells was inhibited by PFT*α*, an inhibitor of P53. Besides, UNC5B-overexpressing cells showed growth rescue in the presence of PFT*α*. Age-related senescence is triggered by P53-P21 or P16 pathways in response to DDR [43, 44]. As such, we speculated that UNC5B induces age-related endothelial cell senescence via the ROS-mediated P53 pathway.

Besides the decline in proliferation potential, senescent cells are characterized by SASP. In this study, we observed that the SASP genes were upregulated in UNC5B-overexpressing cells, while downregulation of UNC5B in senescent cells attenuated the expression of SASP. The SASP is primarily a persistent DDR [45]. The elevated SASP genes induced by UNC5B were further confirmed by our observation that UNC5B induced *γ*-H2AX formation. Therefore, the SASP activated by UNC5B might be attributed to persistent DDR signaling. The effects of UNC5B on SASP activation suggest that UNC5B might exert an effect on various cellular functions in endothelial cells.

Vascular endothelium is a vast endocrine gland of the body, secreting multiple pro-/antiangiogenic factors, cytokines, and a range of biologically active mediators [46, 47]. Previous clinical studies demonstrated that age-related arterial phenotypes are involved the progressive endothelial dysfunction [48, 49]. Impairment of angiogenic capacity is one of the major features of age-related endothelial dysfunction [50]. In our study, overexpression of UNC5B inhibited tube formation in young endothelial cells, while silencing of UNC5B in replicative senescent endothelial cells prevented senescence-associated inhibition of sprouting activity. Moreover, inhibiting UNC5B promoted senescent endothelial cell migration, which is typically associated with angiogenesis. Overexpression of UNC5B impaired the migration of young cells. Thus, we postulated that UNC5B might have antiangiogenic effects. This conclusion was further supported by results from the cell proliferation assay. Consistently, Larrivée and colleagues reported that UNC5B functioned as an antiangiogenic regulator in endothelial cells regulating developmental, postnatal, and pathological angiogenesis [15, 51]. In contrast, Navankasattusas et al. stated a proangiogenic role for UNC5B in placental arteriogenesis [52]. These conflicting findings might be attributed to the different activation states discussed earlier. Regardless, these findings indicate a pivotal role of UNC5B in angiogenesis. Our findings make contribute to clarify the molecular mechanisms of angiogenic impairment capacity in aging, which is essential for discovering novel therapeutic possibilities and prevention methods for age-related vascular diseases.

## 5. Conclusion

In conclusion, we found that UNC5B significantly promoted senescence observed in endothelial cells. Moreover, the ROS-P53 signaling pathway might be a mechanism of UNC5B on senescence. As such, UNC5B might be a potential therapeutic target for endothelial cell senescence and age-related vascular disorders.

## Figures and Tables

**Table 1:** The primer sequences used in this study.

Gene	Forward primer (5′ to 3′)	Reverse primer (5′ to 3′)
GAPDH	TCCAAAATCAAGTGGGGCGA	AAATGAGCCCCAGCCTTCTC
IL-1*α*	AGATGCCTGAGATACCCAAAACC	CCAAGCACACCCAGTAGTCT
IL-1*β*	ATGATGGCTTATTACAGTGGCAA	GTCGGAGATTCGTAGCTGGA
IL-8	TTTTGCCAAGGAGTGCTAAAGA	AACCCTCTGCACCCAGTTTTC
MCP-1	CAGCCAGATGCAATCAATGCC	TGGAATCCTGAACCCACTTCT
ICAM-1	TTGGGCATAGAGACCCCGTT	GCACATTGCTCAGTTCATACACC
P53	CGCTTCGAGATGTTCCGAGA	CTTCAGGTGGCTGGAGTGAG
P21	GTCACTGTCTTGTACCCTTGTG	CGGCGTTTGGAGTGGTAGAAA
UNC5B	GTCGGACACTGCCAACTATAC	CCGCCATTCACGTAGACGAT
VEGFA	AGGGCAGAATCATCACGAAGT	AGGGTCTCGATTGGATGGCA
KDR	GTGATCGGAAATGACACTGGAG	CATGTTGGTCACTAACAGAAGCA

## Data Availability

All data used to support the findings of this study are included within the article. Raw data used to generate the figures are available from the corresponding author upon request.
